# Effectiveness comparisons of Chinese patent medicine on sciatica: A protocol for systematic review and Bayesian network meta-analysis

**DOI:** 10.1097/MD.0000000000023826

**Published:** 2020-12-18

**Authors:** Hongqiang An, Bing Li, Jifeng Zhao, Zhijian Ao, Xiaohui Zhong, Pengfei Zhu, Jianlin Wu

**Affiliations:** a Xintai Affiliated Hospital of Shandong First Medical University; b Shandong University of Traditional Chinese Medicine, Jinan, Shandong Province; c Jinan City Hospital of Integrated Traditional Chinese and Western Medicine; d Changsha City Hospital of Traditional Chinese Medicine; e Zhengxing Hospital of Zhangzhou, Fujian Province; f Linyi Jinluo Hospital, Shandong Province, PR China.

**Keywords:** Chinese patent medicine, network meta-analysis, protocol, sciatica

## Abstract

**Background:**

Sciatica is one of the common clinical diseases. Studies have proved the efficacy of Chinese patent medicine (CPM) in the treatment of sciatica, so far, there has not been a complete systematic review of its effectiveness and safety, and the comparative efficacy and safety of CPM have not been ranked. Therefore, it is necessary to evaluate the efficacy and safety of these CPM by means of systematic review and network meta-analysis (NMA), and to compare them in order.

**Methods:**

We will search PubMed, Cochrane Library, EMbase, Web of Science, CNKI, Wanfang, VIP, CBM and other databases for RCTs of CPM in the treatment of sciatica, (database established until December 30, 2020). In addition, we will manually search the “Pharmaceutical Information”, “National Essential Drug List”, “Chinese Pharmacopoeia”, etc. to inquire about drug instructions, and screen the market circulation and clinically commonly used CPM. We will use RevMan software, gemtc package, GeMTC software for statistical analysis, and draw the surface under cumulative ranking area (SUCRA) to predict the order of curative effect of treatment measures.

**Results:**

Our study will compare and evaluate the effectiveness of CPM in the treatment of sciatica, and rank different CPM. The outcome indicators will include clinical efficacy, pain degree, lumbar spine function and adverse events.

**Conclusion:**

Our research will provide support for clinical practice.

**INPLASY registration number:**

INPLASY2020110073.

## 1 Introduction

Sciatica is a syndrome of pain along the sciatic nerve pathway and distribution caused by primary or secondary damage to the sciatic nerve. As one of the common clinical diseases, it is also one of the more difficult diseases to treat.^[1,2]^According to epidemiological survey, the global prevalence rate of sciatica is 1.2% to 43%, and it mostly occurs in people aged 40 to 60 years.^[3,4]^ This disease can cause paroxysmal, persistent burning and knife-like pain in the distribution area of the sciatic nerve, and it is mostly unilateral. The disease is easy to aggravate at night, which seriously affects the patients health and quality of life. In the United States, conservative treatment of sciatica costs about €30,000 per year on average, and pain seriously affects the quality of life and patients work, resulting in serious socio-economic losses.^[5]^

At present, there are many methods for the treatment of sciatica. The treatment of sciatica in modern medicine mainly includes surgical treatment and conservative treatment.^[6]^ Although surgical treatment can quickly relieve pain and restore the patients function, it has clear surgical indications and the risk of complications, such as injury of nerve root, destruction of spinal stability, injury of dura mater and nerve root, accidental injury of the celiac blood vessels in front of the intervertebral disc and so on. For patients with mild illness, conservative treatment is usually adopted in clinic, which is divided into drug treatment and non-drug treatment. At present, oral or topical non-steroidal anti-inflammatory drugs are routine methods for western medicine to relieve pain. Although the analgesic effect is fast, the drug duration is short, and there are gastrointestinal discomfort, long-term drug resistance and other adverse reactions; Non-drug treatment has little side effects, but the clinical effect is not accurate. Studies of Chinese patent medicine (CPM) in the treatment of sciatica are also reported,^[7,8]^ and the curative effect is exact, but there is a lack of systematic review of CPM in the treatment of sciatica. Furthermore, there are many kinds of CPM, and there is a lack of comparison between the efficacy and safety of them. Therefore, our study will use the network meta-analysis (NMA) for the first time to quantitatively compare the efficacy of different CPM in the treatment of sciatica, so as to select the best intervention measures and provide appropriate theoretical evidence for clinical decision-making.

## 2 Methods

The study will be reported strictly according to the Preferred Reporting Items for Systematic Reviews and Meta-Analysis Protocols (PRISMA-P).^[9]^

### 2.1 Study registration

This NMA has been registered on the International Platform of Registered Systematic Review and Meta-analysis Protocols (INPLASY) and the registration number is INPLASY2020110073 (URL = https://inplasy.com/inplasy-2020-11-0073/).

### 2.2 Inclusion criteria

#### 2.2.1 Type of study

We will include the random control trails (RCTs) of CPM published at home and abroad for the treatment of sciatica, and the language will be limited to Chinese and English.

#### 2.2.2 Participants

We will include patients with clearly diagnosed sciatica according to the diagnostic criteria of sciatica issued by National Institute for Health and Clinical Excellence (NICE).^[10]^ Patients in different intervention groups in the same study will have no significant imbalances in baseline conditions such as age and gender, which is comparable.

#### 2.2.3 Interventions and comparators

The patients in the control group will be treated with routine western medicine, including non-steroidal anti-inflammatory drugs, muscle relaxants, glucocorticoids, nutritional nerve drugs, etc., while the patients in the experimental group will be treated with a kind of CPM or combined with western medicine. The routine therapy between the 2 groups must be the same. The usage, dosage and course of treatment of CMP will be unlimited, and the subjects in both groups will have no additional traditional Chinese medicine (Chinese herbal medicines, Chinese herbal slices, Chinese medicine preparations), massage, acupuncture, surgery or other treatments.

#### 2.2.4 Outcomes

The primary outcome indicator will be the clinical efficacy, which can be divided into 4 levels:

1. Recovery: the pain disappeared completely, straight leg elevation test (-), return to normal work;
2. Significant effect: the pain basically disappeared, the straight leg elevation test (-), but after strenuous exercise, the affected area still feels uncomfortable;
3. Improvement: the pain is significantly reduced, the straight leg elevation test is more than 60 degrees, and the affected area feels sore whenever overworked;
4. Invalid: there is no significant change in the condition after 1 course of treatment.

The secondary outcome indicators will be pain degree and lumbar spine function:

1. Pain degree: Visual Analogue Score (VAS)^[11]^ is a pain evaluation method commonly used in clinical, which is sensitive and easy for patients to judge. Make a table with equal scales from “0” to “10”. A score of “0” indicates that the patient has no pain, and a score of “10” indicates that the patient feels severe pain and is unbearable. The degree of pain gradually increases with the increase of the scale, allowing the patient to select the corresponding scale according to their true feeling of pain before and after treatment, and the tester truthfully records the pain score.
2. Lumbar spine function: Japanese Orthopaedic Association (JOA) Score^[12]^ was created by the Japanese Orthopaedic Association and is a standard for evaluating lumbar spine function. It mainly evaluate patients clinical symptoms, signs and activities of daily living. Patients are scored before and after treatment. The higher the score, the more obvious the recovery of activity function, the lower the score, the worse the recovery of activity.
3. adverse events.

### 2.3 Exclusion criteria

Non-RCT; animal experiment; the object of the study is non-sciatica patients; relevant outcome indicators are not provided in the full text; the same study, repeated publication; patients with poor quality and other diseases.

### 2.4 Search strategy

Two researchers will independently search PubMed, Cochrane Library, EMbase, Web of Science, CNKI, Wanfang, VIP, CBM and other databases for RCTs of CPM in the treatment of sciatica, (database established until December 30, 2020). In addition, manually search the “Pharmaceutical Information”, “National Essential Drug List”, “Chinese Pharmacopoeia”, etc. to inquire about drug instructions, and screen the market circulation and clinically commonly used CPM. Formulate search strategies based on the characteristics of different databases, and use a combination of Medical Subject Headings (MeSH) and free words. Taking Pubmed as an example, the search strategy is shown in Table 1.

**Table 1:** Search strategy for PubMed.

#1	“Sciatica” [Mesh]
#2	“Sciatic Neuralgia” [Title/Abstract] OR “Neuralgia, Sciatic” [Title/Abstract] OR “Neuralgias, Sciatic” [Title/Abstract] OR “Sciatic Neuralgias” [Title/Abstract] OR “Sciatica, Bilateral” [Title/Abstract] OR “Bilateral Sciatica” [Title/Abstract] OR “Bilateral Sciaticas” [Title/Abstract]
#3	#1 OR #2
#4	“Medicine, Chinese Traditional” [Mesh]
#5	“Traditional Chinese Medicine” [Title/Abstract] OR “Chung I Hsueh” [Title/Abstract] OR “Hsueh, Chung I” [Title/Abstract] OR “Traditional Medicine, Chinese” [Title/Abstract] OR “Zhong Yi Xue” [Title/Abstract] OR “Chinese Traditional Medicine” [Title/Abstract] OR “Chinese Medicine, Traditional” [Title/Abstract] OR “Traditional Tongue Diagnosis” [Title/Abstract] OR “Tongue Diagnoses, Traditional” [Title/Abstract] OR “Tongue Diagnosis, Traditional” [Title/Abstract] OR “Traditional Tongue Diagnoses” [Title/Abstract] OR “Traditional Tongue Assessment” [Title/Abstract] OR “Tongue Assessment, Traditional” [Title/Abstract] OR “Traditional Tongue Assessments” [Title/Abstract]
#6	“Yaotong Ning” [Title/Abstract] OR “Shujun Huoxue” [Title/Abstract] OR “Shuangwu Zhentong” [Title/Abstract] OR “Yaoxi Tong” [Title/Abstract] OR “Tongxin Luo” [Title/Abstract] OR “Biqi” [Title/Abstract] OR “Xiatian Wu” [Title/Abstract] OR “Yishen Zhuanggu” [Title/Abstract] OR “Shujin Jianyao” [Title/Abstract] OR “Jingui Shenqi” [Title/Abstract] OR “Renshen Zaizao” [Title/Abstract] OR “Huoluo” [Title/Abstract] OR “Jianbu Zhuanggu” [Title/Abstract] OR “Renshen Yangrong” [Title/Abstract]
#7	#4 OR #5 OR #6
#8	“Placebo” [Title/Abstract]) OR “Randomized” [Title/Abstract]) OR “Randomly” [Title/Abstract]) OR “Trial” [Title/Abstract]) OR “Groups” [Title/Abstract] OR “Randomized Controlled Trial” [Title/Abstract] OR “Controlled Clinical Trial” [Title/Abstract] OR “Randomized Trial” [Title/Abstract]
#9	“Humans” [Mesh] OR “Homo sapiens” [Title/Abstract] OR “Man (Taxonomy) ” [Title/Abstract] OR “Man, Modern” [Title/Abstract] OR “Modern Man” [Title/Abstract] OR “Human” [Title/Abstract]
#10	#3 AND #7 AND #8 AND #9

### 2.5 Study selection and data extraction

Two researchers will independently screen the literatures according to the include and exclusion criteria, and import the literature titles into the Endnote software to check the duplicates, and then eliminate the literatures that do not meet the inclusion criteria by reading the titles and abstracts for preliminary screening; Download and read the full text for re-screening; after determining the final inclusion in the literature, use the pre-designed data extraction table to extract data, and cross-check the results. If there is a difference, the 2 parties will discuss and agree or consult the third party to assist in judgment. The data extraction content will include:

1. Basic literature information: title, first author, publication time, etc.;
2. Basic characteristics of the research object: average age, gender, sample size, intervention measures, treatment course, follow-up time, outcome indicators.

### 2.6 Risk of bias assessment

Two researchers will evaluate the risk of bias in the included studies in accordance with Cochrane Handbook 5.1.0,^[13]^ including:

1. The method of random sequence generation;
2. Whether the allocation plan is hidden;
3. Whether the subject and researcher are blinded;
4. Whether the outcome assessor is blinded;
5. Whether the result data is complete;
6. Whether to report the research results selectively;
7. Other sources of bias.

In case of disagreement, the corresponding author shall make a ruling. According to the results of the evaluation, the Review Manager 5.3 software will be used to make a risk of bias chart for the included studies.

### 2.7 Statistical analysis

RevMan software will be used for bias evaluation and heterogeneity test. Binary variables will use odds ratio (OR) as the effect indicator, and continuous variables will use mean difference (MD) or standardized mean difference (SMD) as the effect indicator, with 95% confidence interval and *P* < .05 used as the standard of statistical difference. The degree of heterogeneity will be judged by I^2^. If *P* ≥ .1 and *I*^2^ ≤ 50%, it means that the heterogeneity between the studies is small, and the fixed effects model will be used, followed by a NMA; if *P* < .1 and *I*^2^ > 50%, we will analyze the source of heterogeneity, use subgroup analysis or sensitivity analysis to deal with obvious clinical heterogeneity, eliminate heterogeneity factors or use random effects model to merge analysis, and use descriptive analysis if the source of heterogeneity cannot be found. Use the R software gemtc package to draw the network diagram. The GeMTC software will be used for NMA and draw the surface under cumulative ranking area (SUCRA). We will use Markov chain-Monte Carlo (MCMC) for Bayesian inference and random effects model for analysis. We will set the initial value, chain number, iteration, annealing, and step length corresponding parameters and calculate. The inconsistency test will adopt the node splitting method. As for *P* > .05 for each study in the subgroup, we will adopt the consistency model, otherwise adopt the inconsistency model. Model convergence will be reflected by potentialscalereduced factor (PSRF). When PSRF is equal to or close to 1, the convergence performance is better and the analysis results of the model are more reliable.

### 2.8 Grading the quality of evidence

We will use the Grading of Recommendations Assessment, Development and Evaluation (GRADE) recommended by Cochrane to evaluate the analysis results.^[13]^ The GRADE evaluation standard evaluates the quality of evidence for each outcome indicator based on five factors: risk of research bias, indirectness of evidence, inconsistency of research results, accuracy of effect estimation, and publication bias.^[14]^ The quality of evidence will be divided into 4 levels: high, moderate, low, very low.

### 2.9 Ethics and dissemination

This study does not involve personal and human trial data and therefore does not require ethical approval.

## 3 Discussion

Sciatica is a common clinical syndrome, which is mainly caused by the pathological changes of the lumbar intervertebral disc, and can cause a serious impact on patients physical and mental health.

CPM have the advantages of multiple targets and multiple pathways in the treatment of sciatica. CPM are made from Chinese medicinal materials and processed through processing, including tablets, granules, ointments, capsules, etc., which are convenient to carry and take. At present, although a number of studies have proved the efficacy of oral CPM in the treatment of sciatica, so far, there has not been a complete systematic review of its effectiveness and safety, and the comparative efficacy and safety of CPM have not been ranked. Therefore, it is necessary to evaluate the efficacy and safety of these CPM by means of systematic review and NMA, and to compare them in order. As far as we know, this is the first time that a systematic review and NMA can be applied to CPM to treat sciatica. However, in specific practice, there may be some defects, such as race, age, gender, and the diversity of medications and dosages, which may lead to some heterogeneity. But, in the end, we hope to provide support for clinical practice.

## Author contributions

**Conceptualization:** Hongqiang An, Bing Li, Jifeng Zhao.

**Formal analysis:** Zhijian Ao, Xiaohui Zhong.

**Funding acquisition:** Jianlin Wu.

**Methodology:** Pengfei Zhu, Jianlin Wu.

**Project administration:** Hongqiang An, Bing Li.

**Software:** Jifeng Zhao, Zhijian Ao, Xiaohui Zhong.

**Writing – original draft:** Hongqiang An.

**Writing – review & editing:** Pengfei Zhu, Jianlin Wu.
